# Second Virtual International Symposium on Cellular and Organismal Stress Responses, September 8–9, 2022

**DOI:** 10.1007/s12192-022-01318-5

**Published:** 2023-01-05

**Authors:** Patricija van Oosten-Hawle, Sarah J. Backe, Anat Ben-Zvi, Dimitra Bourboulia, Mara Brancaccio, Jeff Brodsky, Melody Clark, Giorgio Colombo, Marc B. Cox, Paolo De Los Rios, Frank Echtenkamp, Adrienne Edkins, Brian Freeman, Pierre Goloubinoff, Walid Houry, Jill Johnson, Paul LaPointe, Wei Li, Valerie Mezger, Len Neckers, Nadinath B. Nillegoda, Veena Prahlad, Adam Reitzel, Ruth Scherz-Shouval, Lea Sistonen, Francis T. F. Tsai, Mark R. Woodford, Mehdi Mollapour, Andrew W. Truman

**Affiliations:** 1 Department of Biological Sciences, University of North Carolina at Charlotte, Charlotte, NC 28223 USA; 2 Department of Urology, SUNY Upstate Medical University, Syracuse, New York, NY 13210 USA; 3 Department of Biochemistry and Molecular Biology, SUNY Upstate Medical University, Syracuse, New York, NY 13210 USA; 4 Upstate Cancer Center, SUNY Upstate Medical University, Syracuse, New York, NY 13210 USA; 5 Department of Life Sciences, Ben-Gurion University of the Negev, Beer Sheva, Israel; 6 Department of Molecular Biotechnology and Health Sciences, University of Torino, Turin, Italy; 7 Department of Biological Sciences, University of Pittsburgh, Pittsburgh, PA 15260 USA; 8 British Antarctic Survey, High Cross, Madingley Road, Cambridge, CB3 0ET UK; 9 Department of Chemistry, University of Pavia, Via Taramelli 12, 27100 Pavia, Italy; 10 Border Biomedical Research Center, Department of Pharmaceutical Sciences, University of Texas at El Paso, El Paso, TX 79968 USA; 11 Department of Biological Sciences, University of Texas at El Paso, El Paso, TX 79968 USA; 12 Institute of Physics & Institute of Bioengineering, École Polytechnique Fédérale de Lausanne (EPFL), Lausanne, Switzerland; 13 Urologic Oncology Branch, Center for Cancer Research, National Cancer Institute, Bethesda, MD 20892 USA; 14 Biomedical Biotechnology Research Unit, Department of Biochemistry and Microbiology, Rhodes University, Grahamstown, 6140 South Africa; 15 Centre for Chemico- and Biomedicinal Research, Rhodes University, Grahamstown, 6140 South Africa; 16 Department of Cell and Developmental Biology, University of Illinois Urbana-Champaign, Urbana, IL 61801 USA; 17 School of Plant Sciences and Food Security, Tel-Aviv University, Tel Aviv, Israel; 18 Department of Biochemistry, Temerty Faculty of Medicine, University of Toronto, Toronto, ON M5G 1M1 Canada; 19 Department of Biological Sciences and the Center for Reproductive Biology, University of Idaho, Moscow, ID 83844 USA; 20 Department of Cell Biology, Faculty of Medicine & Dentistry, University of Alberta, Edmonton, Canada; 21 The Department of Dermatology and the USC-Norris Comprehensive Cancer Center, Los Angeles, USA; 22 University of Southern California Keck Medical Center, Los Angeles, CA 90089 USA; 23 CNRS, and Epigenetics and Cell Fate Center, Université Paris Cité, Paris, France; 24 Australian Regenerative Medicine Institute, Monash University, Clayton, VIC Australia; 25 Centre for Dementia and Brain Repair at the Australian Regenerative Medicine Institute, Monash University, Melbourne, VIC Australia; 26 Department of Biology, Aging Mind and Brain Initiative, University of Iowa, Iowa City, IA 52242 USA; 27 Iowa Neuroscience Institute, University of Iowa, Iowa City, IA 52242 USA; 28 Department of Biomolecular Sciences, The Weizmann Institute of Science, Rehovot, Israel; 29 Faculty of Science and Engineering, Cell Biology, Åbo Akademi University, 20520 Turku, Finland; 30 Turku Bioscience Centre, University of Turku and Åbo Akademi University, 20520 Turku, Finland; 31 Departments of Biochemistry and Molecular Biology, Molecular and Cellular Biology, and Molecular Virology and Microbiology, Baylor College of Medicine, Houston, TX 77030 USA

**Keywords:** CSSI symposium, Chaperones, Cancer biology, Heat shock proteins, Hsp70, Hsp90, Proteostasis, Stress responses, Chaperone code

## Abstract

The Second International Symposium on Cellular and Organismal Stress Responses took place virtually on September 8–9, 2022. This meeting was supported by the Cell Stress Society International (CSSI) and organized by Patricija Van Oosten-Hawle and Andrew Truman (University of North Carolina at Charlotte, USA) and Mehdi Mollapour (SUNY Upstate Medical University, USA). The goal of this symposium was to continue the theme from the initial meeting in 2020 by providing a platform for established researchers, new investigators, postdoctoral fellows, and students to present and exchange ideas on various topics on cellular stress and chaperones. We will summarize the highlights of the meeting here and recognize those that received recognition from the CSSI.

## Introduction

**Fig. 1:**
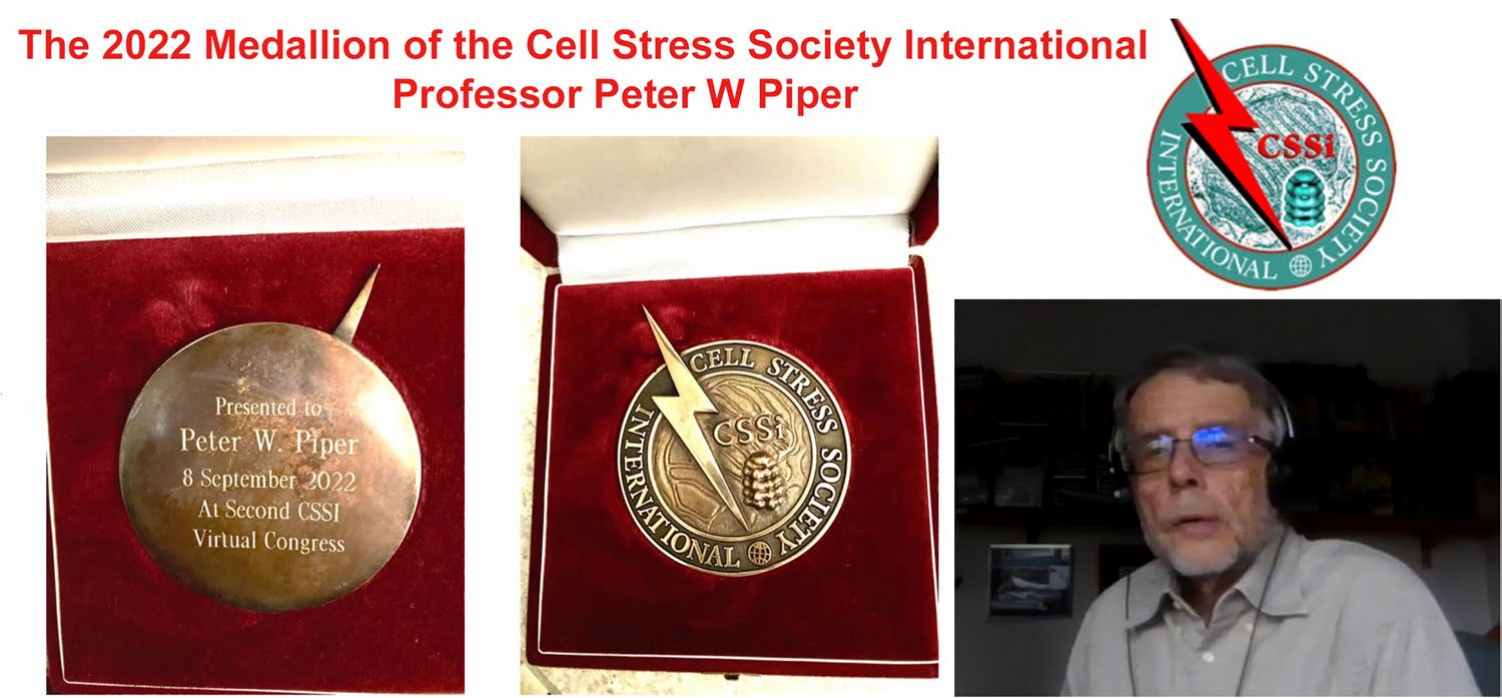
The recipient of 2022 Medallion of the CSSI Professor Peter W. Piper (University of Sheffield, UK)

The Second International Symposium on Cellular and Organismal Stress Responses was attended by over 300 participants from all over the world including locations such as Australia, Israel, and South Africa. The symposium was opened by Mehdi Mollapour followed by Larry Hightower (CSSI Founding President) announcing Professor Peter W. Piper (University of Sheffield, UK) as the recipient of 2022 Medallion of the CSSI (Fig. 1). The Medallion of the Cell Stress Society International is awarded to a senior scientist in recognition of scientific research contributions to the heat shock and cellular stress response field over the course of an entire career. Prof. Piper made major contributions to the understanding of the heat shock response and functional regulation of molecular chaperones such as Hsp90. Valérie Mezger (CSSI-President) and Lea Sistonen (CSSI-President-elect) read the citation and presented the award to Prof Piper. He presented a keynote talk entitled “A foray into the different stress proteins and stress responses of yeast” where he summarized his research work conducted during the past four decades. The entire scientific community congratulates him for this outstanding achievement.

## Cellular stress responses

The first session of the meeting was focused on Cellular Stress Responses, starting with Lea Sistonen (Åbo Akademi University, Turku, Finland) who summarized the results of their recently published work on the stress-type specific transcriptional programs driven by the Heat Shock Factors 1 and 2 (HSF1 and HSF2) (Himanen et al. 2022). The Sistonen lab found that in human cells of epithelial origin, HSF2 is strongly suppressed by TGF-β, which results in destabilized cell–cell adhesion contacts and increased cellular motility. Overexpression of exogenous HSF2 counteracted the TGF-β-induced effects on gene expression and cellular processes of cell–cell adhesion, migration, proliferation, and vasculogenic mimicry. The results suggest that HSF2 can act as an antitumorigenic factor in malignant transformation, thereby stimulating further studies on the context-dependent mechanisms by which HSFs are regulated under physiological and pathological conditions. Valerie Mezger (Epigenetics and Cell Fate Center, Université Paris Cité) presented data showing how brain development responds to prenatal exposure to toxic compunds like alcohol (PAE) and infections which provoke neuroinflammation in the fetal brain and predispose the brain to neurodevelopmental and neuropsychiatric disorders (Schang et al. 2021). She showed that PAE provokes immediate changes in DNA methylation in the mouse developing cortex, which are associated with immediate modifications of gene expression, seem also to persist later in life, and are thus potential biomarkers for the diagnosis and follow-up of at-risk children. Interestingly, stress-responsive transcription factors of the immune/inflammatory pathway (Schang et al. 2021) and HSFs (de Thonel et al. 2022) represent the holes in the defense of the developing brain. They are unexpectedly active and necessary for physiological brain development and are deeply disturbed by prenatal stress. Putative therapeutic treatments should combine subthreshold doses of drugs targeting a range of transcription factors, exclusively in the most vulnerable cell subpopulations, whose identity will be facilitated by single-cell multi-omics approaches.

Len Neckers (National Cancer Institute, Bethesda, USA) revealed how Hsp90 N-terminal inhibitors in combination with Hsp70 can mitigate the compensatory transcriptional activity of HSF1 that has long been held responsible for the lack of single-agent efficacy of Hsp90 inhibitors in the clinic. JG-98, an allosteric Hsp70 inhibitor, blocks Hsp90 inhibitor-dependent induction of Hsp70 at both the mRNA and protein level, with HSF1 protein being degraded or aggregated upon JG-98 treatment. HSF1 is no longer able to initiate Hsp70 compensatory transcription, but the effect of Hsp90 inhibition on its dependent clients is retained. In a bladder cancer mouse model, a combination of JG-98 and the Hsp90 N-terminal inhibitor STA-9090 showed robust and statistically significant tumor growth stabilization as compared to a single agent alone. These data suggest that a combination of both Hsp70 and Hsp90 therapies should be considered to combat the untoward effects of Hsp90 N-terminal inhibition alone and could have promising consequences in the clinic.

## Role of chaperones in disease

Morgana is a ubiquitous Hsp90 co-chaperone, involved in signal transduction (Ferretti et al. 2011). Mara Brancaccio (University of Torino, Italy) recently discovered that Morgana is released from cancer cells and in association with extracellular Hsp90 induces cancer cell migration via Toll-like receptors TLR2, TLR4 and LRP1. Inhibition of Morgana extracellular activity via the use of a blocking antibody inhibits cancer cell migration in vitro and metastasis formation in vivo. In addition, inhibition of Morgana reduced primary tumor growth via macrophage-dependent recruitment of CD8 + T lymphocytes (Poggio et al. 2021; Secli et al. 2021).

The FKBP52 cochaperone is a positive regulator of androgen (AR), glucocorticoid (GR), and progesterone receptor (PR) function and represents an attractive target for the treatment of castration-resistant prostate cancer. Marc B. Cox (University of Texas at El Paso, USA) previously identified MJC13, a first-in-class drug for targeting the regulation of AR. MJC13 works by binding the AR Binding Function 3 (BF3) surface, a putative FKBP52 regulatory surface on AR (De Leon et al. 2011). He has previously demonstrated that FKBP52 directly interacts with β-catenin and promotes β-catenin interaction with AR to synergistically potentiate AR activity (Storer Samaniego et al. 2015). He proposed that FKBP52 and β-catenin act through the AR BF3 surface to allosterically regulate AR activation function 2 (AF2) conformation thereby promoting a distinct AR cistrome and AR-regulated transcriptional program. He assessed the AR-dependent cistrome and transcriptome by ChIP-seq and RNA-seq in the presence or absence of hormone in wild-type or *fkbp52*-deficient 22Rv1 cells. In addition, an FKBP52 interactome analysis was conducted by tandem affinity purification followed by mass spectrometry in 22RV1 cells. The number of unique AR DNA binding sites in the presence of hormone increased from approximately 1800 to over 20,000 in the *fkbp52*-deficient cells suggesting a potential dysregulation of AR DNA binding specificity in the absence of FKBP52. Pathway analysis of differentially expressed genes demonstrated dysregulation of oxidative phosphorylation pathways in *fkbp52*-deficient 22Rv1 cells. Consistent with these findings, members of the peroxiredoxin family, implicated in oxidative stress protection and resistance to treatment, are among the top FKBP52 protein interactors.

Castration-resistant prostate cancer (CRPC) presents a persistent health risk and is the second leading cause of cancer-related death in American men. AR is a key regulator of prostate cancer and the principal target of current prostate cancer treatment collectively termed androgen deprivation therapies (ADT). Hsp70 has emerged as a promising target in the treatment of CRPC because of the chaperone’s role in stabilizing AR. Inhibition of Hsp70 with JG-98 results in potent growth suppression of the CRPC model cell line 22Rv1 and resensitized 22Rv1 to ADT drugs enzalutamide and abiraterone. Surprisingly, this observation was independent of JG-98’s impact on AR protein levels and rather was related to a previously unrecognized impact on mitochondrial proteostasis. Frank Echtenkamp (NCI, USA) showed that JG-98 permeated mitochondria where it disrupts mitochondrial Hsp70 activity by preventing association with the nucleotide exchange factor, GrpEL1. This results in a rapid decline in mitochondrial ribosomal subunits, inhibition of mitochondrial translation, and suppression of mitochondrial respiration. Direct suppression of mitochondrial respiration with the complex I inhibitor, IACS-010759, was also able to resensitize 22Rv1 to ADT drugs. Their work has uncovered a previously unrecognized metabolic vulnerability of CRPC cells that may be able to extend the efficacy of currently available prostate cancer therapies.

Nadinath Nillegoda (Monash University, Melbourne, Australia) presented beautiful data clarifying how disaggregases are regulated in vivo. He showed that selective assembly of an Hsp70-DNAJA1-DNAJB1 complex during the late phase of stress recovery allows refolding proteins that have co-aggregated with cellular condensates. When activated, this disaggregase provides resistance to stress toxicity and contributes to amyloid disposal. Strikingly, Hsp70-DNAJA1-DNAJB1 disaggregase is one of the earliest protein quality control machines to collapse in cells undergoing replicative aging (Mathangasinghe et al. 2022). Overall, his fascinating work establishes a critical interface between protein disaggregation, cell repair, and senescence.

## Structure and function of stress proteins

The determination of a protein’s structure and its interactions at the molecular level can provide substantial mechanistic insight into a protein’s function. In the Structure and Function of Stress Proteins session (chaired by Sarah Backe), Francis Tsai (Baylor College of Medicine, Houston, Texas) presented novel findings on the function of the N-terminus of Hsp100. Hsp100 unfoldases are ubiquitously found in unicellular microbes (e.g., ClpA/B/C/X and Hsp78/104) as well as in mitochondria of animal cells (i.e., CLPX and SKD3) and usually facilitate either the disaggregation of aggregated, stress-damaged proteins, or the degradation of misfolded polypeptides. We show that the N-domain of Hsp100 disaggregases can directly bind polypeptides and becomes crucial for protein disaggregation by the yeast Hsp104-Hsp70:Hsp40 bi-chaperone system when the Hsp70 system is absent. Interestingly, the *Leishmania* parasite that requires Hsp100 for stage differentiation has evolved its Hsp100 N-domain to adapt to the highly oxidizing environment inside host macrophages. The 1.06-Å crystal structure of the Hsp100 N-domain from *L. mexicana* revealed a network of methionine-aromatic amino acid interactions that both stabilizes and protects the N-domain from oxidative damage (2), presenting a potential drug target for the development of new anti-leishmaniasis.

Walid Houry (University of Toronto, Toronto, Canada) identified a new component of the R2TP chaperone complex, the Deleted in Primary Ciliary Dyskinesia (DPCD) protein. R2TP is a highly conserved chaperone complex formed by two AAA + ATPases, RUVBL1 and RUVBL2, that associate with PIH1D1 and RPAP3 proteins (Seraphim et al. 2022; Zhao et al. 2005) R2TP functions with several other chaperones including Hsp90 and Hsp70 to promote macromolecular complex formation (Lynham and Houry 2022). Hsp90-R2TP-DPCD acts to regulate ciliogenesis through modulating signaling pathways. His findings highlight a new function for Hsp90-R2TP in maintaining cellular protein homeostasis.

Jill Johnson (University of Idaho, Idaho, USA) used a series of yeast Hsp90 mutants that appear to disrupt either the “loading”, “closing”, or “reopening” steps in the Hsp90 folding cycle to dissect how Hsp90 and co-chaperones cooperate during the folding cycle. Her data indicates that Hch1 acts to destabilize Hsp90-nucleotide interaction, hindering formation of the closed conformation, whereas Cpr6 counters the effects of Hch1 by stabilizing the closed conformation. Hch1 and the homologous Aha1 share some in vivo functions, but the role of Hch1 in inhibiting progression through the early stages of the folding cycle is unique. Sensitivity to the Hsp90 inhibitor NVP-AUY922 also correlates with the conformational cycle, with mutants defective in the loading phase being most sensitive and those defective in the reopening phase being most resistant to the drug. Mark Woodford (SUNY Upstate Medical University, Syracuse, NY, USA) discussed the role of post-translational modifications on the TRAP1 protein. TRAP1 is an ATP-dependent molecular chaperone that suppresses mitochondrial respiration, thereby regulating global metabolic flux. His work showed that TRAP1 is subject to O-GlcNAcylation following mitochondrial import. This modification suppressed TRAP1 ATPase activity while increasing binding to the inhibitor Ganetespib. Further, TRAP1 loss significantly attenuated cellular response to O-GlcNAcase inhibitors (Kim et al. 2022), suggesting a reciprocal regulatory mechanism for TRAP1-GlcNAc and contributing to our understanding of the chaperone code (Backe et al. 2020).

Brian Freeman (University of Illinois, Urbana-Champaign, Illinois, USA) presented his group’s findings on the physical interactome of the Hsp90 molecular chaperone. Using the nonnatural amino acid p-benzoyl-L-phenylalanine (Bpa), which is a photoactivatable crosslinker, in conjunction with large-scale mass spectrometry they captured and identified over 1000 proteins in close association with Hsp90. The Hsp90 interactors functioned in a wide variety of cellular pathways including protein folding and transport. Follow-up studies by the Freeman research team showed that Hsp90 has an active role in supporting the fidelity of ribosome initiation. Significantly, disruption of this Hsp90 function triggers a heat shock response, which has long been correlated with Hsp90 inhibitors. Overall, the presented work suggests Hsp90 has a broad capacity to interact with and regulate numerous native proteins to support the proteostasis process. Paolo De Los Rios (École Polytechnique Fédérale de Lausanne, Lausanne, Switzerland) provided novel findings on J-Domain Proteins (JDPs), the major cochaperone regulators of Hsp70. JDPs display a staggering diversity in domain architectures (besides the ubiquitous and namesake J-domain), functions, and cellular localization. Despite their essential role in cellular proteostasis, only a few JDPs have been well-studied. Presently, the number of JDP sequences identified by means of efficient sequencing techniques has exponentially increased, beyond the ability of careful manual curation. Malinverni et al. (Malinverni et al. 2022) have recently provided a broad view of the JDP repertoire accessible from public databases and used it then for an automated classification scheme, based on Artificial Neural Networks (ANNs), showing that the sequences of the J-domains carry sufficient information to recover with high reliability the phylogeny, localization, and domain composition of the corresponding full-length JDP. The interpretability of the ANNs allowed the discovery of the sequence positions that contribute the most to the classification, finding that they are localized at the interaction interface between the J-domain and Hsp70, hinting at a strong coevolution of J-domains with their obligatory Hsp70 partners, necessary to build chaperone circuits for specific functions.

## Organismal stress responses

In the Organismal Stress Response session, chaired by Veena Prahlad (University of Iowa, USA), Adrienne Edkins (Rhodes University, Makhanda, South Africa) showed data on how extracellular Hsp90 is important in cell migration and invasion processes. Hsp90 alters both the levels and morphology of the matrix by interacting with the matrix client protein fibronectin (FN). In cell line models of FN fibrillogenesis, extracellular Hsp90 promoted FN matrix assembly, but FN mutants with reduced Hsp90 interaction failed to promote assembly. Consistent with this, C-terminal but not N-terminal Hsp90 inhibition promoted matrix turnover. This turnover required the catabolic FN receptor LRP1, which is also a receptor for extracellular Hsp90, potentially suggesting the presence of an extracellular Hsp90-FN-LRP1 ternary complex (Chakraborty et al. 2020). Together, these data identify Hsp90 in the extracellular environment as an important regulator of matrix stability (Chakraborty and Edkins 2021). Following the theme on extracellular Hsp90 (eHsp90), Wei Li (University of Southern California Keck Medical Center, Los Angeles, USA) looked at the identification of natural “wound healing driver genes” to facilitate the development of new and effective therapeutics. When the skin is injured, there is a massive and time-dependent deposition of eHsp90α protein into the wound bed, but the precise role of the eHsp90α protein remained elusive. The recent establishment of Hsp90α knockout (KO) mice which are phenotypically indistinguishable from their wild-type counterparts provides an animal model to directly address whether or not eHsp90α is a wound-healing driver gene. In this system, full-thickness skin wound closure is significantly delayed. The quality of healed wounds is compromised, especially in the thickness of the epidermis and extracellular matrix organization in the dermis. Similarly, bleomycin-induced lung injury is more severe and recovery slower in Hsp90α-KO mice. Topical application of recombinant eHsp90α protein or its F-5 fragment promotes wound closure in Hsp90α-KO mice as effectively as Hsp90α-wt mice. Thus, their study provides direct support for eHsp90α as a potential driver for normal wound healing in different organs (Tang et al. 2022). Anat Ben-Zvi (Ben-Gurion University of the Negev, Beer Sheva, Israel) used bioinformatic analysis to understand expression patterns of the basal chaperone system across human tissues. The data revealed that the expression levels of most chaperones show tissue-specific changes and that related human tissues presented similar chaperone expression patterns that were conserved in evolution. This organization connects the chaperone system to the cellular proteome, suggesting that the varied expression of chaperones determines its capacity and versatility (Shemesh et al. 2021). They further explored whether differentiation transcription factors that regulate protein expression can also drive chaperone expression to meet the cell-specific proteome folding requirements. Using HLH-1, the MyoD ortholog in *Caenorhabditis elegans* that specifies muscle fate was examined. Ectopic expression of HLH-1 in embryos induced the selective expression of chaperones with HLH-1 occupancy sites at their promoters. Disrupting the consensus sequence in muscle chaperone promoter reporters abolished their expression. Consistently, *hlh-1* knockdown in embryos and larvae disrupted muscle proteostasis and reduced the expression of some muscle chaperones. Taken together, it is likely that differentiation can remodel the conserved cell-specific chaperone network to meet the cell’s needs (Nisaa and Ben-Zvi 2022).

## Evolution and stress responses

Adam Reitzel (UNC Charlotte, USA) chaired the Evolution and Environmental Stress Session. Pierre Goloubinoff (Tel-Aviv University, Tel Aviv, Israel) presented work focusing on how Hsp70 protects cells against protein aggregation. His lab generated a reporter chaperone substrate, MLucV, composed of a heat-sensitive luciferase core, flanked by heat-resistant fluorescent domains, which upon denaturation formed a discrete population of small dodecameric, round-shaped aggregates, held together by a core of misfolded luciferases, wrapped by native fluorophores at the surface. Combining FRET and enzymatic activity measurements provided new details on distinct MLucV conformations: native, unfolded, Hsp70-bound, and aggregated. The chaperone mechanism first involved DnaJ-binding to the stable preformed aggregates, followed by Hsp70 binding and ATP-fueled disaggregation and unfolding. This stretched the MLucV polypeptides beyond the urea-unfolded state. GrpE addition, which released the HSP70, lead to the spontaneous refolding of MLucV to the native state. Iterative ATP-fueled unfolding-refolding cycles by the Hsp70 unfoldase could accumulate native MLucV species even under elevated temperatures that are unfavorable to the native state. The results thus clearly exclude the so-called holdase activity of Hsp70 and DnaJ, from taking a significant part in the catalytic, ATP-fueled mechanism by which stable protein aggregates become actively and iteratively converted into metastable native proteins under nonequilibrium conditions highly detrimental to the native state (Tiwari et al. 2022). Melody Clark (British Antarctic Survey, Cambridge, UK) looked at the resilience to environmental stress in the Greenland blue mussels, a highly timely subject relating to the effects of climate change. Summer temperatures, which can reach 36 °C in the intertidal is beyond the upper thermal limits of the blue mussel, *Mytilus edulis.* Transcriptomic analyses on samples collected directly from the natural environment revealed a homogeneous gene expression pattern, indicating *M. edulis* to have the capacity to withstand the current rates of Arctic warming. This lack of transcriptional signal in animals varying by 24 °C was almost certainly due to their acclimation to a fluctuating temperature regime, which has increased their thermal tolerances. More in-depth analysis of the transcriptome data revealed a massive expansion of the HSPA12 gene. Overall these data suggest that this expanded gene family act as intertidal regulators underpinning thermal resilience (Clark et al. 2021).

## Targeting molecular chaperones with novel small molecules

Several diseases linked to protein aggregation and toxic cell stress responses can be ameliorated by genetic overexpression or chemical activation of select chaperones, such as Hsp70. Dimitra Bourboulia (SUNY Upstate Medical University, USA) chaired this session and introduced Jeff Brodsky (University of Pittsburgh, USA). He presented data on the identification of small molecule Hsp70 activators that target the chaperone’s ATP binding domain and mimic the action of Hsp40 cochaperones, which augment Hsp70 function. As hypothesized, one of these activators, MAL1-171, reduced the levels of protein aggregates in several disease models. Subsequent rounds of medicinal chemistry and analysis identified more drug-like compounds that retained the ability to activate Hsp70 and—as hypothesized—also decreased the concentration of disease-associated protein aggregates in vitro (Chiang et al. 2019). Moreover, MAL1-271 was able to reduce oxidative stress and prolong the life of zebrafish in a model for acute kidney injury (AKI), a malady that affects ~ 50% of all patients hospitalized in the ICU. Current efforts were then described which are being devoted to investigating this compound and other “proteostasis modulators” in a new inducible AKI mouse model, one in which the gene encoding the GRP170 chaperone in the nephron can be targeted and deleted (Porter et al. 2022). Inhibition of Hsp90 with ATP-competitive antagonists drives the semiselective degradation of proteins that are dependent on this chaperone for their stability. In cancer, mutated gene products are often highly dependent on Hsp90 for their folding. Paul LaPointe (University of Alberta, Canada) revealed the potential of Hsp90 inhibitors to drive the degradation of these mutated gene products and enhance the presentation of neoantigenic peptides on Type I Major Histocompatibility Complexes. He has found that Hsp90 inhibition increases levels of HLA-B and HLA-C MHCI and leads to enhanced recognition of these cells by T-cells in both a mouse colon cancer model and a human tumor organoid system. He has also found that Hsp90 inhibition leads to large-scale changes in the immunopeptidome. Peptides derived from known Hsp90 interactors are enriched after Hsp90 inhibition. Hsp90 inhibitors may be useful to enhance immune checkpoint blockade inhibition therapy in the clinic (Mercier and LaPointe 2022). Giorgio Colombo (University of Pavia, Italy) presented new results on computational studies of Hsp90. In particular, he showed new approaches, based on the analysis of atomistic molecular simulations, to characterize the dynamic states of the chaperone and correlate them with the modulation of the protein’s functional activity. The data generated are currently being used to design new chemical tools to interfere with Hsp90 pathologic activities (D'Annessa et al. 2020; Paladino et al. 2020).

## Rapid fire session

This year, CSSI organized a rapid-fire session to promote work from undergraduate and graduate students as well as postdoctoral researchers. There were outstanding presentations from the following; Sara Sannino (Brodsky lab), Elham Ahanin (Mollapour lab), Kevin Daupin (Mezger lab), Jonathan Hibshman (Goldstein lab), Dovile Milonaityte (van Oosten-Hawle lab), Sehee Min (Prahlad Lab), Gregory Blatch (Blatch lab), Natasha Hockaden (Carpenter lab), Ainella Rysbayeva (Truman lab), Federica Scalia (Capello lab), Laura Wengert (Mollapour lab), and Silvina Beatriz Nadin (Nadin lab).

## Awards

The Executive Council of the CSSI awarded The Ferruccio Ritossa Early Career Award to Andrew Truman (UNC Charlotte, USA) for his contributions to CSAC as a senior editor and his work understanding the posttranslational modifications (PTMs) on Hsp70. He described recent findings on this chaperone-client code including novel mass spectrometry strategies to understand how PTMs may influence chaperone interactions in yeast and mammalian cells (Nitika et al. 2020, 2022). The Alfred Tissières Young Investigator Award was awarded to Ruth Scherz-Shouval (Weizmann Institute of Science, Israel) for her outstanding work on roles of HSF1 in cancer. She presented data demonstrating that HSF1 mediates the transcriptional program of cancer-associated fibroblasts (CAFs) resulting in the remodeling of the extracellular matrix and the content of exosomes. Finally, Larry Hightower presented service awards to the following new CSSI Fellows; Manuela Truebano (University of Plymouth, UK) and Lingling Chen (Indiana University Bloomington, USA). CSSI Senior Fellows; Barbara Lipinska (University of Gdansk, Poland), Michael Evgen’ev (Institute of Molecular Biology RAS, Russia), Patrick Arrigo (Université Claude Bernard Lyon, France), Cristina Bonorino (Universidade Federal de Ciências da Saúde de Porto Alegre, Brazil), Willem van Eden (Utrecht University, The Netherlands), Michael Freeman (Vanderbilt University, USA), Richard D. Mosser (University of Guelph, Canada), Ryan D. Martinus (University of Waikato, New Zealand), George Perdrizet (The Hospital of Central Connecticut, USA), and Steven Witkin (Cornell University Medical College, USA). The following speakers won best prize for rapid fire presentation; 1st place, Sara Sannino (University of Pittsburgh, USA) “Proteostasis adaptation is fundamental for cancer cell survival during proteotoxic stress”; 2nd place Kevin Daupin (CNRS, France) “Innovative models to explore the contribution of the HSF2 stress pathway in a neurodevelopmental disorder model”; Jonathan Hibshman, University of North Carolina Chapel Hill (USA) “Tardigrade small heat shock proteins can limit heat- and desiccation-induced protein”; and 3rd place Emily E. Hirsch (University of Iowa, USA) “Examining the developmental role of the HSP110/70/40 chaperone complex in C. elegans”, Yaa Amankwah (Miami University, USA) “Grp94 works upstream of BiP in protein remodeling under heat stress”, and Elham Ahanin (State University of New York, USA) “Pharmacological inhibition of the co-chaperone Protein Phosphatase-5 activates the extrinsic apoptotic pathway in kidney cancer”.
